# MiRNA-based therapeutic potential in multiple sclerosis

**DOI:** 10.3389/fimmu.2024.1441733

**Published:** 2024-08-29

**Authors:** Ana Zabalza, Agustin Pappolla, Manuel Comabella, Xavier Montalban, Sunny Malhotra

**Affiliations:** ^1^ Vall Hebron University Hospital & Research Institute (VHIR), Multiple Sclerosis Centre of Catalonia (Cemcat) & Neurology Department, Universitat Autonoma de Barcelona, Barcelona, Spain; ^2^ Faculty of Medicine, University of Vic - Central University of Catalonia (UVicUCC), Vic, Spain

**Keywords:** microRNAs, multiple sclerosis, neurology, biomarkers, therapeutic targets

## Abstract

This review will briefly introduce microRNAs (miRNAs) and dissect their contribution to multiple sclerosis (MS) and its clinical outcomes. For this purpose, we provide a concise overview of the present knowledge of MS pathophysiology, biomarkers and treatment options, delving into the role of selectively expressed miRNAs in clinical forms of this disease, as measured in several biofluids such as serum, plasma or cerebrospinal fluid (CSF). Additionally, up-to-date information on current strategies applied to miRNA-based therapeutics will be provided, including miRNA restoration therapy (lentivirus expressing a specific type of miRNA and miRNA mimic) and miRNA inhibition therapy such as antisense oligonucleotides, small molecules inhibitors, locked nucleic acids (LNAs), anti-miRNAs, and antagomirs. Finally, it will highlight future directions and potential limitations associated with their application in MS therapy, emphasizing the need for improved delivery methods and validation of therapeutic efficacy.

## 1 Introduction

MicroRNA (miRNAs) are crucial in regulating gene expression, mainly operating via post-transcriptional mechanisms that may influence various physiological processes. Multiple sclerosis (MS) is a chronic immune-mediated disorder of the central nervous system (CNS). During the last decade, there has been a crucial advancement in MS etiology and treatment. However, effective management remains challenging, especially for progressive forms of MS, which motives us to explore novel therapeutic strategies.

This review will cover the current knowledge of miRNAs in MS and explore their role in disease pathogenesis as biomarkers with therapeutic potentials. Furthermore, we will examine novel miRNA-based therapeutic approaches, such as miRNA restoration and inhibition therapies, highlighting their potential in MS therapeutic arsenal. Finally, we will address the challenges and future direction of miRNA-based therapies in MS, emphasizing the utmost need for overcoming barriers to clinical translation.

## 2 MicroRNAs

miRNAs are small, single stranded, non-coding RNA molecules ranging in size from 18 to 24 nucleotides long, with an average length of 22 nucleotides. They play a crucial role in post-transcription regulation of gene expression by binding to complementary sequences in the messenger RNA (mRNA), leading to either mRNA degradation or inhibition of its translation into protein (1).

**Figure 1:**
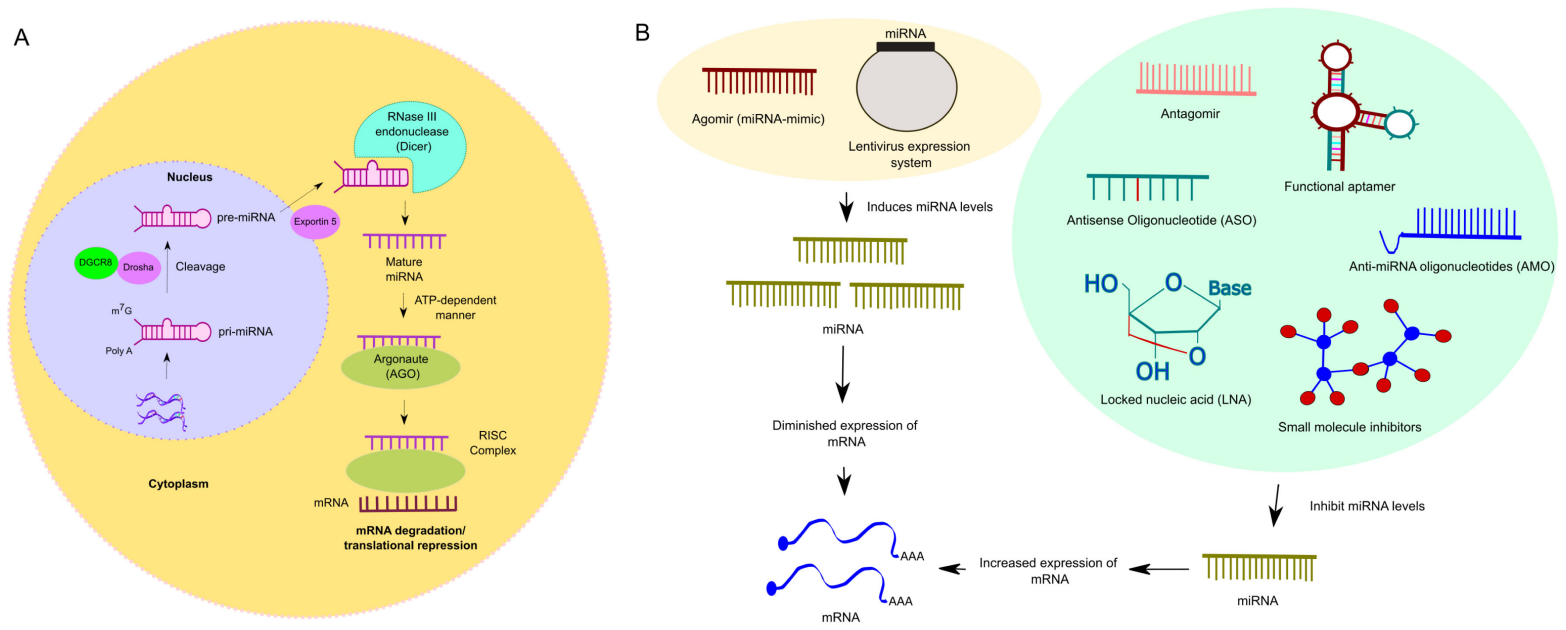
**(A)**. Overview of miRNA biogenesis (Canonical). The process initiates within nucleus, where RNA polymerase II (RNAPII)-dependent transcription produces a capped and polyadenylated transcript termed primary miRNA (pri-miRNA). The pri-miRNA undergoes processing mediated by the Drosha, an RNase III endonuclease, alongside its cofactor, DGCR8, yielding smaller stem-looped structures termed precursor miRNA (pre-miRNA). These pre-miRNAs are eventually exported from the nucleus by their transporter, Exportin5, into the cytosol. Once pre-miRNAs reach the cytosol, a RNase III endonuclease (Dicer) further processes the pre-miRNA, resulting in mature miRNA. As a subsequent step, the mature miRNA then integrates into the miRNA-induced silencing complex (miRISC). In this step, the mature miRNA interacts with the complementary sequences predominantly situated in the 3’-untranslated regions (3’-UTRs) of mRNA, leading to post-translational gene silencing. **(B)**. Overview of blocking and activation strategies to modulate miRNA expression: Blocking miRNA expression can be achieved through various methods such as antisense oligonucleotides (ASOs), small molecule inhibitors, locked nucleic acid (LNA), anti-miRNA oligonucleotides (AMO), aptamers, and antagomirs. ASOs, which are single-stranded oligodeoxynucleotides, bind to RNA and prevent its attachment to the ribosome or block protein translation. Small molecule inhibitors regulate post-transcriptional expression of disease-associated genes, potentially reversing dysfunctional pathways. LNAs, chemically modified RNA nucleotides, exhibit specific binding and stability. AMOs, complementary to miRNA sequences, prevent miRNA from interacting with its target mRNA. Aptamers, synthetic oligonucleotides, bind specific target molecules and can prevent miRNA-mRNA interactions or induce miRNA degradation. Antagomirs, synthetic RNA oligonucleotides, block miRNA expression by forming stable duplexes or preventing their interaction with target mRNAs, sometimes inducing miRNA degradation. However, activation or restoration therapy involves using miRNA mimics, such as Agomirs. These are double-stranded and chemically modified to improve cellular uptake and stability, mimicking the function of mature endogenous miRNAs. Additionally, shRNA lentivirus expression systems efficiently overexpress specific miRNAs. Both approaches are utilized in gain-of-function studies.

miRNAs are transcribed from DNA sequences into primary miRNAs transcripts (pri-miRNAs). This transcription is followed by a cleavage process mediated by two components: DiGeorge Syndrome Critical Region 8 (DGCR8) in complex with Drosha (2, 3). Once complexed, these components cleave the pri-miRNAs, producing precursor miRNAs (pre-miRNAs). The two-nucleotide 3’ overhang of pre-miRNAs is bound by a complex composed of Exportin5 and RanGTP, which facilitates the pre-miRNA export and further processing to produce mature miRNA duplexes. Once pre-miRNAs reach the cytosol, they are processed by the RNase III endonuclease (Dicer), resulting in miRNAs (4). These strands can then bind to Argonaute (AGO) proteins in an ATP-dependent manner, forming a miRNA-induced silencing complex (miRISC) (2). Once formed, the RISC complex interacts with mRNA, leading to either degradation or translational repression (5). **Figure 1A** illustrates the canonical miRNA biogenesis of miRNAs. Some miRNAs are generated from spliced introns known as mirtrons, representing a non-canonical miRNA biogenesis. Mirtrons bypass the Drosha processing step and enter the miRNA maturation pathway directly after splicing (6).

miRNAs are classified based on their genomic origin and sequence similarities. The nomenclature includes a unique identifier number (e.g., miR-146) and letter suffixes in cases of closely related miRNAs (e.g., miR-146a, miR-146b). This system helps distinguish miRNAs with similar sequences but different genomics origins or functions (7). When two distinct loci in the genome produce identical mature miRNA, an additional number is assigned after the miRNA’s name to distinguish them. For example, mir-92a-1” and “mir-92a-2” refer to two different precursor miRNAs that both give rise to the same mature miRNA, “miR-92a.” These precursors are encoded by separate genes located in different regions of the genome (8). Furthermore, the directionality of the miRNA strand determines the name of the mature miRNA. The strand arising from the 5’ end of the pre-miRNA is designated as the 5p miRNA, while the strand arising from the 3’ end is designated as the 3p miRNA (2).

miRNAs exist in two forms in various body fluids, such as in serum, plasma or cerebrospinal fluid (CSF): freely circulating forms, known as cell-free miRNAs (cf-miRNAs), and those encapsulated within exosomes, termed exosomal miRNAs (exo-miRNAs) (9). Cell-free miRNA circulates in the body fluid, often bound to proteins such as Argonaute 2, which protect them from degradation. On the other hand, exosomal miRNAs are enclosed within exosomes, small vesicles secreted by cells. These exosomes retain the membrane signature of the host cell, including specific proteins and lipids that reflect the cell of origin. Therefore, exosomal miRNA serves as biomarker, indicating cellular source and potential disease states (10). Exosomes are the smallest type of extracellular vesicles, with an average diameter of 30 to 150nm. They are formed through the endosomal pathway and play a significant role in cell-to-cell communication by transporting proteins, lipids, and miRNAs between cells, thereby influencing various physiological and pathological processes (11). Furthermore, miRNAs could be transported through the bloodstream bound to high density lipoprotein, which can deliver these miRNAs to various tissues, including the brain (12).

## 3 Multiple sclerosis pathogenesis and therapeutic arsenals

MS is a chronic immune-mediated disease affecting the CNS. Although its exact etiology remains unknown, it is believed to result from a combination of genetic and environmental factors, such as past Epstein Barr virus (EBV) infection, tobacco exposure, or low vitamin D (13–16).

Pathologically, MS is characterized by both inflammatory and neurodegenerative components. The inflammatory component, associated with MS relapses, begins with the pathological activation of autoreactive lymphocytes against CNS antigens in predisposed individuals, leading to the clonal proliferation of these cells. Upon crossing the blood-brain barrier (BBB), these cells initiate an inflammatory cascade resulting in CNS demyelination and axonal degeneration. The initial CNS damage activates microglia, which secrete cytokines and chemokines, facilitating the entry or activation of diverse immune cell types – including B cells, T cells (CD4 and CD8), dendritic cells (DC) or monocytes-macrophages into the CNS. The neurodegenerative component, which contributes to the progressive phase of the disease, is characterized by a chronic CNS inflammation resulting in dysfunction of neuronal networks, inadequate mechanism of repair, and chronic neurodegeneration. Microglia and astrocytes play pivotal roles in this neurodegenerative component (17).

In 80-85% of people with MS, the disease begins with an acute episode referred to as clinically isolated syndrome (CIS). Natural history studies indicate that patients suffer successive clinical attacks, known as relapsing remitting MS (RRMS), and over time, incomplete recovery from each episode, leading to the accumulation of persistent symptoms. Eventually, approximately 15-65% of patients transition to sustained neurological deterioration, referred to as secondary progressive MS (SPMS). Patients with primary progressive MS patients (PPMS) are those who develop a progressive disability worsening from the beginning with or without superimposed relapses (10-15%) (18). Currently, there are no clear mechanistic differences between these clinical forms, and the phenotype classification is under revision (19). The diagnosis of MS is confirmed by a combination of biological, clinical and radiological criteria (McDonald criteria 2017) (20).

There is currently no cure for MS. However, numerous immunomodulatory and immunosuppressive disease-modifying therapies (DMTs) have exhibited diverse levels of effectiveness in reducing clinical relapse rates, radiological activity and short-term disability progression when administered during the relapsing phase of the disease (17, 21). Early DMT treatment has shown to decrease the number of patients who transition to a progressive phase (22, 23). Approved treatments for relapsing forms can be categorized based on their efficacy into low-moderate efficacy treatments (interferons, teriflunomide, glatiramer acetate, and fumarates), high-efficacy agents (like S1P receptor modulators, and cladribine) and very high efficacy treatments (anti-CD20 monoclonal antibodies, natalizumab, and alemtuzumab). Ocrelizumab is the only approved treatment for PPMS patients, demonstrating a moderate reduction in disease progression (24–26).

Currently, various tools are being utilized to evaluate disease activity and treatment response in MS. Brain and spinal cord magnetic resonance imaging (MRI) has proven effective as a biomarker for assessing focal inflammation. However, MRI measures have also shown reliability in predicting long-term disease progression by evaluating brain T2 lesion volume (27), paramagnetic rim lesions (PRLs) (28) and CNS atrophy (29). In addition to imaging techniques, ongoing research is focusing on evaluating routine diagnostics and prognostics serum or CSF biomarkers. Some biomarkers are gaining attention, including Chitinase 3-like 2 (CHI3L2), neurofilament light chains (NfL) and glial fibrillary acidic protein (GFAP), as they hold potential additional value in predicting disease progression (30, 31).

## 4 Multiple sclerosis and cell-free miRNAs

Significant strides have been made in our understanding of cell-free miRNAs in MS over the past decade. Studies highlight the advantages of these easily accessible biomarkers for potential clinical use (32).

Several studies have reported changes in serum miRNA levels in MS patients compared to HC. In recent study observed a negative correlation between serum levels of miR-92a-3p and miR-486-5p and the number of lesions in the posterior inferior lobule and lateral temporal cortex. Additionally, miR-142-5p levels were positively correlated with the functional connectivity strength between the temporal lobe and the retrosplenial cortex, indicating a link to functional and structural neuroimaging outcomes in MS patients (33). Serum levels of miR-24-3p, miR-128-3p and miR-191-5p were found to be elevated in MS patients compared to healthy controls. After further classification of MS patients into RRMS and PPMS patients, miR-191-5p and miR-24-3p, showed significant differences compared to healthy controls. In the case of miR-128-3p, the differences remain significant for PPMS compared to HC. Additionally, miR-24-3p correlated with disability progression, while miR-128-3p correlated with the annual relapse rate in RRMS (34). miR-223, miR-23a, and miR-15b showed decreased serum levels in MS patients when compared to healthy controls (HC). Furthermore, miR-223 and miR-15b exhibited higher diagnostic potential for discriminating between PPMS patients and HC, as assessed by ROC curve analysis (35).

The expression of miRNAs in CSF has been studies extensively. A study has identified downregulated expression of CSF miR-143-3p and let-7b-5p in patients with PPMS compared to other neurological disease (OND). Additionally, miR-26a-5p was downregulated in patients with PPMS when compared to RRMS patients. miR-142-5p expression was upregulated in patients with RRMS compared to OND. Similarly, CSF miR-142-5p shows a positive correlation with clinical progression (higher number of T2 lesions) (36). Interestingly, the absence of CSF miR-219, a factor known to regulate oligodendrocyte maturation, was associated with MS (37).

Studies on inflamma-miRs (a subset of miRNAs involved in regulating inflammatory processes) have provided insights into inflammatory processes in the disease (38). One study found elevated levels of miR-125a-5p and miR-34a in the plasma of RRMS patients as compared to healthy controls (39). Conversely, circulating miR-146a-5p, were diminished in RRMS patients when compared to HC (39). Regarding active MS MRI lesions, increased levels of miR-146a/b and miR-21 were associated with the presence of Gd+ lesions in MS patients, correlating with the number of Gd+ lesions and neurofilaments light (NF-L) levels (40). In another study, the serum miR-126-3p was significantly downregulated in RRMS patients in baseline and during NTZ treatment when compared to HC. However, it was significantly increased in one patient who developed progressive multifocal leukoencephalopathy (PML) (41). In another study conducted by Regev et al. (2017), the correlation between miRNAs and MRI was investigated using the T1/T2 ratio, which assesses the destructive potential of lesions and brain atrophy. The study found significant correlations between miRNAs and MRI measures, indicating protective roles for four miRNAs (hsa-miR-142-5p, hsa-miR-143-3p, hsa-miR-181c-3p, and hsa-miR-181c-5p) and pathogenic roles for others (hsa-miR-375 and hsa-miR-629-5p with brain atrophy, hsa-miR-486-5p and hsa-miR-92a-3p with the T1/T2 ratio). However, after multiple comparisons, no association remained significant (42). Finally, a larger serum cohort was used in a study where the authors found that patients with benign MS had lower levels of miR-25-3p and higher levels of miR-320b. Elevated levels of miR-320b were associated with the development of secondary progressive multiple sclerosis (SPMS). Additionally, brain parenchymal fraction, an indicator of neurodegeneration, was found to correlate negatively with miR-25-3p and positively with miR-320b (43).

Regarding therapeutic considerations, limited data exists in the field of miRNAs. A study by De Vito F et al., 2022 found that patients with MS treated with dimethyl fumarate (DMF) who had high miR-142-3p levels in their CSF exhibited higher disease activity and were more frequently switched to high efficacy treatment compared to those with low levels. Furthermore, CSF levels of miR-142-3p were found to correlate with IL-1β signaling and neuronal excitability in patients with MS. In the same line, IL-1β/miR-142-3p axis was implicated in synaptopathy in an experimental autoimmune encephalomyelitis (EAE) model. Interestingly, fumarate treatment was observed to suppress miR-142-3p, thereby attenuating this axis and its associated synaptopathy (44). Natalizumab (NTZ) modulates the miR-126-3p expression and its target genes such POU2AF1 and Spi-B (45). Patients treated with dimethyl fumarate (DMF) showed reduced expression of circulating plasma miR-146a-5p, miR-125a-5p, and miR-155 after 4 months of treatment. However, miR-146a-5p and 125a-5p were the only miRNAs related to disease progression as measured by EDSS and ARMSS scores after 12 months of treatments as compared to non-progressed patients (39). In a study conducted by Gonzalez-Martinez A et al., researchers confirmed miR-548a-3p as a biomarker for monitoring treatment response in MS patients treated with fingolimod. Higher serum miR-548a-3p were observed in NEDA-3 (no evidence of disease activity) patients as compared to EDA-3 (evidence of disease activity). They observed that miR-548a-3p exhibited a strong ability to predict which patients achieved NEDA-3 at the 2-year in the validation group. These results suggest that miR-548a-3p has potential as a useful tool for assessing treatment response in clinical settings for individuals with MS (46).

In summary, research has revealed various cell-free miRNAs may have implication in the pathogenesis of MS, including miR-92a-3p, miR-486-5p, miR-24-3p, miR-128-3p, let-7b-5p, miR-143-3p, miR-34a, miR-126-3p, miR-125a-5p, miR-25-3p and miR-320b. Reduced miR-223, miR-23a and miR-15b levels showed high diagnostic prospect. Differential CSF miRNA expression (e.g miR-142-5p) correlated with clinical progression. DMTs have unveiled associations between miRNAs expression levels- such as miR-142-3p, miR-125a-5p, miR-155, and miR-146a-5p- and treatment response to DMF. Furthermore, miR-548a-3p expression has been associated with treatment response to fingolimod.

## 5 Multiple sclerosis and exosomal microRNA

Significant progress has been made in the study of exosomal miRNAs in MS. A recent study identified miR-18a-5p, Let-7g-5p, miR-374a-5p and miR-145-5p as having both pro- and anti-inflammatory actions. Notably, miR-342-3p and miR-150-5p exhibited anti-inflammatory properties. These miRNAs were significantly upregulated in both serum-derived exosomes and CSF of patients with RRMS compared to HC (47).

In EAE animal model of MS, researchers observed that exosomes derived from bone marrow mesenchymal stem cells (BMSCs), termed BMSC-exos, contained miR-367-3p. Co-culture of these exosomes with microglia resulted in a significant reduction of microglial ferroptosis by downregulating Enhancer of zeste homolog 2 (EZH2) and upregulating the expression of SLC7A11, which ultimately ameliorated the severity of EAE *in vivo*. This study suggests that overexpression of miR-367-3p holds promise as a potential therapeutic strategy for EAE (48).

Furthermore, exosomes derived from T cells of MS patients exhibited elevated level of miR-326, particularly in patients with RRMS compared to HC. MiR-326 is implicated in the immunopathogenesis of MS by promoting TH17 differentiation and maturation, suggesting its potential as a diagnostic and prognostic biomarker (49). Another study highlighted the abundance of plasma let-7i in circulating exosomes, which inhibits the differentiation of regulatory T (Treg) cells in patients with MS. This inhibition reduces the expression of IGF1R and TGFBR1 on naïve CD4+ T cells, leading to a decrease in Treg cell frequency in MS (50). Additionally, a study identified a panel of nine serum miRNAs, including miR-374a-5p, miR-15b-5p, miR-30b-5p, miR-223-3p, and miR-342-3p, that were upregulated in RRMS patients as compared to progressive form of the disease. Conversely, miR-432-5p, miR-433-3p, miR-485-3p and miR-23a-3p were found to be downregulated in RRMS patients as compared to progressive forms of MS (51) as summarized in **Table 1**.

**Table 1:** Selected miRNA-based biomarkers in multiple sclerosis.

miRNA	Biomarker type	Source	Main findings
Cell free miRNA			
*↑miR-92a-3p, miR−486-5p*,	Prognostic	Serum	Positive correlation with the number of matter lesion volumes in cervical spine (33)
*↓miR−142-5p*	Prognostic	Serum	Negative correlation with disease duration (33)
*↑miR-24-3p, miR-128-3p*	Prognostic	Serum	Positive correlation of disability accumulation and disease activity, Respectively (34)
*↓miR-15b, miR-223*	Diagnostic	Serum	Discriminate between PPMS patients from HC (35)
*↑miR-142-5p*	Diagnostic	CSF	Positive correlation with clinical progression as measured by higher number of T2 lesions (36)
*↓miR-146a-5p*	Diagnostic	Plasma	Diminished levels in RRMS patients compared to HC (39)
*↑miR-21, miR-146a/b*	Diagnostic	Plasma	Discriminates between MS patients with and without Gd+ and positively associated with the number of Gd+ lesions and NF-L levels (40)
*↑miR-320b,↓miR-25-3p*	Prognostic	Serum	High levels of miR-320b correlate positively, while low levels of miR-25-3p correlate negatively, with BPF (43)
*↑miR-142-3p*	DMT Response	CSF	Higher levels of miR-142-3p is associated with higher disease activity and frequently switched to high-efficacy treatment (44)
*↓miR-126-3p*	Diagnostic	Serum	miR-126-3p was significantly downregulated in RRMS patients at baseline and during NTZ therapy as compared to HC (41)
*↓miR-125a-5p, miR-146a-5p*	DMT Response	Plasma	Downregulated after 4 months of DMF treatment and related to disability progression (39)
miRNA	Biomarker type	Source	Main findings
Exosomal miRNA			
*↑miR-548a-3p*	DMT Response	Serum	Higher miR-548a-3p levels were observed in NEDA-3 patients compared to EDA-3 patients treated with fingolimod (46)
*↑miR-150-5p*	Diagnostic	Serum	miRNA levels were significantly upregulated in RRMS patients compared to HC (47)
*↑miR-326*	Diagnostic	T cells	Elevated levels of miR-326 in RRMS patients compared to HC (49)
*↑let-7i*	Diagnostic	Plasma	Elevated level of let-7i in MS patients as compared to HC (50)
*↑miR-15b-5p,↑374a-5p*, *↑30b-5p,↑342-3p, ↑223-3p*, *↓23a-3p,↓433-3p,↓485-3p, ↓432-5p*	Diagnostic	Serum	Nine miRNAs discriminate with RRMS as compared to progressive forms of the disease (51)
*↑miR-367-3p*	Therapeutic	BMSC-Exos	Reduction in microglial ferroptosis by repressing EZH2, which improves clinical status of EAE (48)

PPMS, Primary progressive multiple sclerosis; HC, healthy control; RRMS, relapsing-remitting multiple sclerosis; Gd+, gadolinium-enhanced; BPF, brain parenchymal fraction; NF-L, neurofilament light chain; DMF, dimethyl fumarate; NTZ, natalizumab; EZH2, enhancer of zeste homolog 2; NEDA3, no evidence of disease activity 3; EDA3, evidence of disease activity 3.

↑, upregulated; ↓, downregulated.

In summary, these studies elucidated the role of miRNAs, including let-7g-5p, let-7i, miR-18a-5p, and miR-326, in pathogenesis of MS. Moreover, distinct miRNAs panel shows promise for differentiating between clinical forms of MS.

## 6 Future directions and potential limitations of miRNAs in MS therapy

MiRNAs offer several advantages as biomarkers for disease and treatment response monitoring. They exhibit high stability in body fluids such as serum, plasma, and CSF samples. The use of miRNAs facilitates non-invasive techniques for sample collection.

Several techniques can be utilized depending on whether we intend to inhibit or restores the expression of miRNAs. miRNA expression can be restored by two ways. Efficient and long-term overexpression of specific miRNAs, revealing complex regulatory interactions including target-directed miRNA degradation beyond seed sequences, can be achieved using shRNA lentivirus expression systems (52). Another approach in miRNAs could be restored involves miRNAs mimics. MiRNA mimics are chemical compounds designed to replenish miRNAs that are downregulated in diseases, effectively mimicking the activity of endogenous miRNAs (53).

An approach to blocking miRNAs expression includes antisense oligonucleotides (ASOs), small molecule inhibitors, LNAs, anti-miRNAs oligonucleotides (AMO), aptamers, and antagomirs (**Figure 1B**).

ASOs are single-stranded oligodeoxynucleotides that can modify RNA via complementary base-pairing. ASOs can bind to RNA (pre-mature) and prevent its attachment to the ribosome or directly block protein translation (54). MiRNAs-targeted small molecule inhibitors for disease treatment can regulate the post-transcriptional expression of disease-associated genes. Dysfunctional pathways can be reversed by correcting dysregulated gene expression (55). LNAs are chemically modified RNA nucleotides that alter ribose by connecting 2´oxygen and 4´carbon with an extra bridge. LNAs are the newest RNA analogs that exhibit specific binding, stability, and nuclease resistance (56).

Furthermore, AMOs are designed to be complementary to the sequence of miRNAs. Once AMOs enter the cells, they hybridize with the miRNAs, forming a duplex. This prevents the miRNAs from interacting with its target. AMOs can sequester the miRNAs, thereby blocking its ability to bind to mRNA molecules. Some AMOs are designed to induce the degradation of miRNAs molecules by recruiting RNAse H or other nucleases (57, 58). Aptamers are short, synthetic, single-stranded oligonucleotides comprised of either RNA or DNA that bind to specific target molecules. They typically range in length from 20 to 100 nucleotides (59). RNA aptamers can act as specific targeting molecules to regulate allosteric modulations in the processing of pri-miRNAs. Aptamers can prevent the interaction of miRNAs with its mRNA target. Additionally, specific aptamers are engineered to induce the degradation of miRNAs molecules, reducing miRNAs levels and activity. This targeted approach selectively modulates miRNAs regulation and presents new avenues for developing novel therapies (60).

Finally, antagomirs are synthetic 2-O- methyl RNA oligonucleotides designed to block the miRNAs expression. Their size varies from 17 to 22 nucleotides in length (61). In their mechanism of action, antagomirs are similar in concept to another class of molecules called anti-miRNAs. They block the expression of miRNAs by forming stable duplexes with miRNAs or by preventing their interaction with target mRNAs. In certain cases, they can also induce miRNAs degradation (58).

To date, some miRNAs therapeutics have been in clinical trials for Hepatitis C (NCT02452814), wounds (NCT03603431), and non-small-cell lung cancer (NCT02369198), among other conditions (62).

Potential applications of miRNAs should focus on identifying molecules related to demyelination and remyelination and investigating their therapeutic potential to develop strategies for promoting neurorepair and reducing neuroinflammation in MS.

Challenges associated with miRNAs research include identifying the most promising miRNAs candidates for therapeutic applications in MS. This challenge arises primarily due to the limited sample sizes often encountered in miRNAs studies, making it unclear whether observed effects are consistent and reproducible across larger populations. Robust validation through larger-scale studies or meta-analyses is essential to confirm the therapeutic potential of identified miRNAs. Another significant complication in miRNAs therapeutics is the mode of delivery. Many current delivery methods face barriers in efficiently transporting miRNAs across biological barriers such as the BBB in the case of MS. Overcoming the delivery challenges is crucial to ensure therapeutic miRNAs can reach the CNS and exert their intended effects. However, there is a high need to minimize variability by implementing standardized protocols both intra and inter lab environment. Optimization of normalization methods is crucial to ensure accurate representation of biological conditions. Additionally, rigorous application of quality control protocols is essential to mitigate sources of error and enhance the predictive power of miRNA-based assays.

Additionally, achieving CNS-specific targeting is essential for miRNAs therapeutics to minimize off-target effects and maximize efficacy. Designing delivery systems or modifying miRNAs to enhance CNS specificity can help tailor treatments to the disease sites while minimizing adverse effects on healthy tissues. Moreover, addressing toxicity-related concerns is vital for the safe and effective use of miRNAs therapeutics. It is crucial to ensure that therapeutic miRNAs do not elicit harmful immune responses or undesired side effects, which is crucial for clinical translation. Furthermore, off-target effects, where miRNAs inadvertently modulate unintended gene expression, pose another challenge that must be solved. Designing miRNAs mimics or inhibitors with improved specificity can help mitigate off-target effects and may improve the precision of miRNA-based therapies in MS. The stability of therapeutic miRNAs during storage and delivery is another critical consideration that needs to be addressed. Developing strategies to protect miRNAs from degradation and maintain their activity over time is essential for ensuring the efficacy of miRNAs-based treatments.

In summary, while miRNAs therapeutics hold significant promise for treating various diseases, including MS, addressing the challenges of candidate selection, delivery, specificity, toxicity, off-target effects, and stability is essential for realizing their full potential in clinical applications by developing new therapies in MS.
